# Integrated experimental and computational study reveals enhanced antimicrobial efficacy of amoxicillin loaded in oleic acid nanoemulsion against multidrug-resistant *Salmonella typhimurium*

**DOI:** 10.1039/d5ra05196g

**Published:** 2025-09-19

**Authors:** Seifeldin Elabed, Amira K. Mohamed, Hassan Youssef, Huda Abdelhamied, Eyad Mokhtar, Wael M. Elshemey, Medhat W. Shafaa, Ayman Meselhi

**Affiliations:** a Medical Biophysics Division, Physics Department, Faculty of Science, Helwan University Cairo Egypt seifEldin25@science.helwan.edu.eg AmiraKhaledMohamedMohamed515st@science.helwan.edu.eg Hassan.fathi@science.helwan.edu.eg eiadmokhtar67@gmail.com shafaa@science.helwan.edu.eg ameselhy@science.helwan.edu.eg; b Physics Department, Faculty of Science, Islamic University of Madinah Madinah Kingdom of Saudi Arabia welshemey@iu.edu.sa

## Abstract

The escalating crisis of antimicrobial resistance (AMR) poses a severe threat to global health, demanding innovative strategies to rejuvenate conventional antibiotics against multidrug-resistant (MDR) pathogens like *Salmonella typhimurium*. This study presents an integrated experimental and computational investigation into an oleic acid-based nano-emulsion designed to restore amoxicillin's potency. The amoxicillin-loaded nano-emulsion, prepared *via* spontaneous emulsification and ultrasonication, yielded stable, semi-spherical nanoparticles with a mean hydrodynamic diameter of 199.6 nm, a low polydispersity index (0.331), and a high negative zeta potential of −46.3 mV, ensuring excellent colloidal stability. *In vitro* antimicrobial testing against MDR *S. Typhimurium* revealed a profound 2.3-fold enhancement in antibacterial activity; the nano-formulation produced an inhibition zone 133% larger (35.0 ± 2.1 mm) than free amoxicillin (15.0 ± 1.8 mm). To elucidate the molecular underpinnings, molecular docking and 100 ns molecular dynamics simulations confirmed the stable, high-affinity binding of amoxicillin within the catalytic cleft of its target enzyme, Penicillin-Binding Protein 3 (PBP3a). The interaction, with a strong binding affinity of −9.4 kcal mol^−1^, was energetically driven by dominant van der Waals (−27.1 kcal mol^−1^) and electrostatic (−80.6 kcal mol^−1^) forces, yielding a total binding free energy of −32.0 ± 8.0 kcal mol^−1^*via* MM-PBSA analysis. Crucially, *in silico* ADMET predictions forecast a paradigm shift in pharmacokinetics, projecting a 132 000-fold increase in intestinal absorption and a 95-fold reduction in hepatotoxicity risk. These integrated findings provide a robust, mechanistically detailed rationale for using oleic acid nano-emulsions as a powerful delivery platform to overcome bacterial resistance.

## Abstract 2

Oleic acid nanoemulsion undergoes pH-dependent self-assembly in acidic infection sites, disrupting bacterial membranes and enhancing amoxicillin uptake. This lowers MIC, accelerates bacterial killing, and inhibits biofilm formation. Boosting β-lactam efficacy against Salmonella typhimurium.

## Graphical abstract

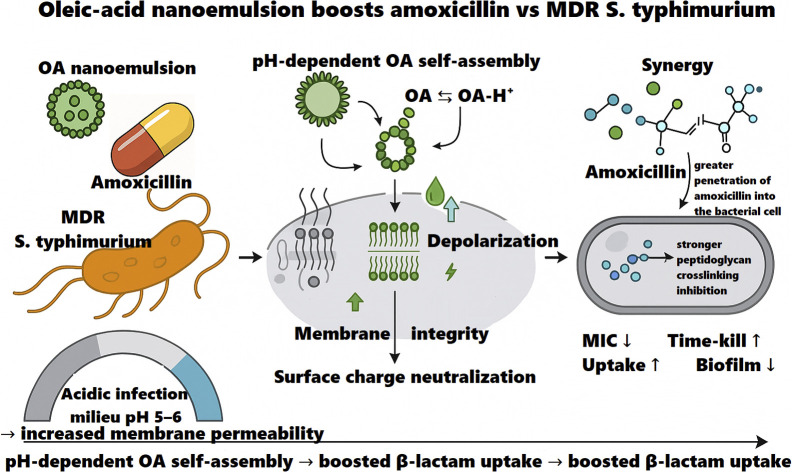

## 1. Introduction

Antimicrobial resistance (AMR) has advanced from a looming concern to an immediate threat. The 2024 WHO GLASS report documents a continued rise in bloodstream isolates resistant to three or more antibiotic classes, with carbapenem-resistant *Klebsiella pneumoniae* now exceeding 70% of isolates in several intensive-care settings.^1^ Updated global burden modelling attributes 1.27 million deaths directly to bacterial AMR in 2019 and 4.95 million when associated causes are included.^2^ Without decisive intervention, the seminal O'Neill review still projects up to 10 million AMR-related deaths annually by 2050.^3^

The economic stakes mirror the human toll. World Bank macro-economic simulations predict that unchecked AMR could shave 1.1–3.8% from global GDP by 2050–an annual loss of US $1 trillion to US $3.4 trillion.^4^ At the bedside, Canada's national assessment estimated that each resistant infection adds about C$18 000 in hospital costs, foreshadowing a cumulative health-system burden of more than C$120 billion by mid-century.^10^

Misuse and over-use of antimicrobials remain the dominant selection pressures. Agriculture alone consumes roughly 73% of the global antibiotic supply–about 63 000 metric tons each year–largely for growth promotion and prophylaxis in livestock.^5^ Horizontal gene-transfer vehicles such as conjugative plasmids, integrons and transposons accelerate the dissemination of resistance determinants far beyond the pace of chromosomal mutation.^6^

*Salmonella enterica* serovar *Typhimurium* illustrates the clinical complexity of AMR pathogens. Non-typhoidal *Salmonella* causes an estimated 93.8 million episodes of gastroenteritis and about 155 000 deaths each year, while invasive disease results in more than half a million bloodstream infections worldwide.^7^ Surveillance across Europe shows that multidrug-resistant *S. typhimurium* now accounts for almost 30% of human isolates, and fluoroquinolone non-susceptibility has approached 20% since 2018.^8^ In sub-Saharan Africa, where invasive non-typhoidal salmonellosis disproportionately affects young children, case-fatality rates reach 25% when effective therapy is unavailable.^9^ β-Lactam antibiotics such as amoxicillin are still frontline agents, yet the spread of β-lactamase-producing strains is eroding clinical success, lengthening hospital stays and compounding economic losses.

Oleic acid exhibits pronounced pH-dependent self-assembly: at high pH (≥10) the oleate form organizes as micelles (CMC ∼0.1 mM at pH 12), whereas in the pH ∼7.5–9 range vesicles and worm-like micelles can coexist. This behavior rationalizes the nanoemulsion's colloidal stability and supports a dual role for OA as both carrier matrix and membrane-active co-agent.^86–88^

The innovation pipeline is critically thin: the 2023 WHO analysis lists only 97 antibacterial candidates in clinical phases, and fewer than a dozen possesses genuinely novel modes of action against priority pathogens.^11^ This paucity has shifted attention toward delivery strategies that can re-arm existing drugs. Oil-in-water nano-emulsions formulated with GRAS excipients have shown superior membrane penetration, protection from enzymatic degradation and sustained release, reducing minimum inhibitory concentrations four- to eight-fold against multidrug-resistant Gram-negative bacteria.^12,14^ Broader nanotechnology platforms–polymeric micelles, lipid nanoparticles and metallic carriers–exhibit complementary mechanisms that disrupt biofilms and bypass efflux pumps.^13^ Multiscale molecular-dynamics simulations now allow rational design of such nanocarriers, predicting drug–carrier interactions and release kinetics before expensive laboratory work.^15^

We propose that an oleic-acid nano-emulsion will fortify amoxicillin against multidrug-resistant *Salmonella typhimurium* by boosting delivery, membrane passage and shielding it from enzymes. The study will compare nano-loaded and free drug *via* agar well diffusion, while formulating the emulsion through spontaneous emulsification and ultrasonication. Physicochemical traits—particle size, PDI, zeta potential and morphology—will be characterized, encapsulation verified spectroscopically, and antimicrobial gains quantified statistically from inhibition-zone diameters relative to standard control groups.

## 2. Materials and methods

### 2.1 Nanoparticle physicochemical properties

#### 2.1.1 Bacterial strain and culture conditions

*Salmonella typhimurium* ATCC BAA-190 was acquired from the American Type Culture Collection (ATCC) and cultured on Mueller–Hinton agar (MHA) plates according to CLSI guidelines.^16^ The bacterial strain was maintained at −80 °C in 20% glycerol stocks to preserve viability and genetic stability. For each experimental procedure, a fresh aliquot was revived by overnight incubation in tryptic soy broth (TSB) at 37 °C with continuous shaking at 200 rpm. The bacterial suspension was subsequently adjusted to 0.5 McFarland turbidity standard (approximately 1.5 × 10^8^ CFU per mL) using a McFarland densitometer to ensure standardized inoculum density across all assays.^17^

#### 2.1.2 Chemicals and reagents

High-purity amoxicillin trihydrate (≥98% purity) was procured from Sigma-Aldrich (St. Louis, MO, USA) and stored under desiccated conditions at 4 °C to prevent degradation. Oleic acid (≥99% purity) and polysorbate-80 (Tween-80) were obtained from the same supplier and used as received without further purification. Mueller–Hinton broth and agar were purchased from Oxoid Ltd (Basingstoke, UK). All aqueous solutions were prepared using ultrapure water (18.2 MΩ cm resistivity) generated by a Milli-Q water purification system. Other reagents including sodium chloride, potassium phosphate buffer components, and organic solvents were of analytical grade and procured from certified suppliers.^18^

#### 2.1.3 Preparation of amoxicillin nano-emulsion

The amoxicillin-loaded nano-emulsion was prepared using a modified spontaneous emulsification technique coupled with high-energy ultrasonication.^19,20^ Initially, 1.0 g of amoxicillin trihydrate was dissolved in 25 mL of deionized water containing 1.6% (v/v) Tween-80 as the primary surfactant. The aqueous phase was stirred magnetically at 1000 rpm for 15 minutes to ensure complete dissolution and homogenization. Subsequently, oleic acid (5% v/v) was added dropwise to the aqueous phase under continuous magnetic stirring at 1000 rpm for 5 minutes to form a coarse pre-emulsion. The pre-emulsion was immediately subjected to probe ultrasonication using a Vibra-Cell VCX 750 ultrasonic processor (Sonics & Materials Inc., Newtown, CT, USA) operating at 20 kHz frequency and 50% amplitude for 10 minutes. The sonication was performed in an ice bath to prevent thermal degradation of the antibiotic and minimize solvent evaporation. Following ultrasonication, the nano-emulsion was filtered through a 0.22 μm sterile polyethersulfone membrane filter to remove any large aggregates and ensure sterility. The final formulation was stored at 4 °C in amber glass vials to protect from light and maintained under sterile conditions until use.^21^

#### 2.1.4 Physicochemical characterization

Amoxicillin is a small amphoteric β-lactam (MW = 365 Da; *c* Log *P* ≈ 0.75; p*K*_a_ values ∼3–9) with milligram-per-milliliter aqueous solubility near neutral pH. As a non-surface-active small molecule, AMX is not expected to form micelles or DLS-detectable aggregates in water. Moreover, DLS/ζ-potential are poorly suited to sub-nanometer solutes and provide limited, often misleading information in this regime. Accordingly, we did not pursue DLS/ζ of free AMX; instead, we report DLS/ζ only for the nanoemulsion where colloidal droplets are present.^89–92^

The hydrodynamic diameter, polydispersity index (PDI), and zeta potential of the nano-emulsion droplets were determined using dynamic light scattering (DLS) with a Zetasizer Nano ZS instrument (Malvern Panalytical, Worcestershire, UK).^22^ Measurements were conducted at 25 °C using disposable polystyrene cuvettes for size analysis and folded capillary cells for zeta potential determination. Each sample was appropriately diluted (1 : 100 v/v) with ultrapure water to minimize multiple scattering effects and ensure accurate measurements. The refractive index of the dispersed phase was set to 1.46 (oleic acid) and the dispersant to 1.33 (water). Data acquisition involved automatic optimization of measurement parameters, with each result representing the average of three consecutive measurements from independent nano-emulsion batches. Morphological analysis was performed using transmission electron microscopy (TEM) with a JEOL JEM-2100 instrument operating at 120 kV acceleration voltage. Samples were prepared by placing a drop of diluted nano-emulsion onto carbon-coated copper grids, followed by negative staining with 1% phosphotungstic acid solution and air drying prior to examination.^23^

Spectroscopic confirmation of amoxicillin encapsulation was achieved using a Shimadzu UV-2600 spectrophotometer equipped with a deuterium lamp for UV region analysis. Absorption spectra were recorded over the wavelength range of 200–600 nm using quartz cuvettes with 1 cm path length. The scanning parameters included a resolution of 0.1 nm and a scanning speed of 240 nm min^−1^. Samples analyzed included free amoxicillin solution, blank nano-emulsion, and amoxicillin-loaded nano-emulsion, all appropriately diluted to fall within the linear range of the Beer–Lambert law. The characteristic absorption maximum of amoxicillin at approximately 229 nm was monitored to confirm successful drug loading and to assess any spectral shifts indicative of drug–excipient interactions.^24^

#### 2.1.5 Antibacterial activity assessment and statistical analysis

The antimicrobial efficacy of the nano-formulated amoxicillin was evaluated against *Salmonella typhimurium* using the standardized agar well diffusion method in accordance with Clinical and Laboratory Standards Institute (CLSI) guidelines M100-S34.^25^ Mueller–Hinton agar plates were uniformly inoculated with a bacterial suspension adjusted to 0.5 McFarland standard (approximately 1.0 × 10^6^ CFU per mL), and wells of 6 mm diameter were loaded with 100 μL of test solutions including amoxicillin-loaded nano-emulsion, free amoxicillin (100 μg mL^−1^), and blank nano-emulsion as controls. After 24 hours of incubation at 37 °C, inhibition zone diameters were measured in millimeters using calipers, with twelve independent measurements per treatment group across four replicate plates to ensure statistical robustness. Statistical analyses were implemented using two complementary approaches: primary analysis in R software^26^ employed the Wilcoxon Rank–Sum Test for comparing inhibition zone diameters between treatment groups, with robustness validated through permutation testing (10 000 permutations, random seed 123) and data visualization performed using the tidyplots package.^27^ Secondary analysis utilized Python libraries where data were expressed as mean ± standard deviation computed with NumPy,^55^ variance homogeneity was assessed using Levene's test,^56^ and group comparisons were performed using two-tailed Welch's *t*-tests when variances differed, implemented in SciPy.^57,58^ Confidence intervals incorporated Welch-Satterthwaite degrees of freedom corrections,^59^ effect sizes were calculated using Cohen's *d*, and statistical significance was set at *α* = 0.05 throughout all analyses.^60^

### 2.2 Computational methodology

#### 2.2.1 Structural identification

The primary amino acid sequence of *Salmonella typhimurium* FtsI (peptidoglycan D,d-transpeptidase) was obtained from the UniProt database (Q7CQD7|Q7CQD7_SALTY). Conserved domains and active-site motifs were identified using InterProScan 5, which integrates multiple signature databases (*e.g.*, Pfam, SMART).^28^

#### 2.2.2 Molecular docking

The three-dimensional structure of amoxicillin (PubChem CID 33613) was retrieved from the PubChem database. The structure was converted to PDB format using Open Babel,^29^ and its energy was minimized using the MMFF94 force field to generate a low-energy conformation.^30^ AutoDockTools 4 was used to assign Gasteiger partial charges and define rotatable bonds for the ligand.^31^

The three-dimensional structure of FtsI was obtained from the AlphaFold Protein Structure Database (AlphaFold ID: Q7CQD7). Preparation involved removing crystallographic water molecules, adding polar hydrogens, and assigning Kollman charges using AutoDockTools.^31^ A grid box of 24 × 24 × 24 Å^3^ was defined, centered on the catalytic region (including the active site residue at position 300) identified through conserved active-site residue analysis from the Structural Identification Stage.

Rigid-body docking was performed using GNINA, with an exhaustiveness parameter set to 16, generating 20 binding modes.^32^ Rigid docking was deemed appropriate based on the typically limited backbone flexibility observed in class A PBPs, where ligand binding often induces only minor loop movements (≤1 Å).^33^ The top five poses, ranked by binding affinity, were retained, and the conformation with the best score (kcal mol^−1^) was selected for subsequent analysis. Protein–ligand interactions for this pose were characterized in 3D using the Protein–Ligand Interaction Profiler (PLIP)^34^ and visualized in 2D using Discovery Studio 2025.^35^

#### 2.2.3 Molecular dynamics simulations and MM-PBSA analysis

All-atom molecular dynamics (MD) simulations were performed with GROMACS 2022.4.^36,37^ The protein was modeled using the CHARMM36 force field,^38^ and amoxicillin was modeled with CHARMM General Force Field (CGenFF) parameters.^39^ The complex was solvated in a cubic box with the TIP3P water model,^40^ keeping a 10 Å buffer from the edges. The system was neutralized and brought to a 0.15 M ionic strength with Na^+^ and Cl^−^ ions.

Energy was minimized using the steepest-descent algorithm until the maximum force was below 1000 kJ mol^−1^ nm^−1^. The system was then equilibrated for 100 ps in the NVT ensemble (300 K, velocity-rescaling thermostat^41^) and 100 ps in the NPT ensemble (1 atm, Parrinello–Rahman barostat^42^). A 100 ns production MD run was performed with a 2 fs time step. Long-range electrostatics were handled by the Particle Mesh Ewald (PME) method with a 10 Å cutoff,^43^ and frames were saved every 10 ps. System stability was confirmed by analyzing RMSD, RMSF, radius of gyration, and hydrogen bonds, with equilibration reached around 10 ns.

Binding free energies (Δ*G*_bind_) were calculated using the Molecular Mechanics Poisson–Boltzmann Surface Area (MM-PBSA) method *via* the g_mmpbsa tool.^44^ The energy was determined by the equation:

End-state binding free energies (Δ*G*_bind_) were calculated using the Molecular Mechanics Poisson–Boltzmann Surface Area (MM-PBSA) method as implemented in the g_mmpbsa tool.^44^ The binding free energy (Δ*G*_bind_) was calculated using the equation:Δ*G*_bind_ = *G*_complex_ − (*G*_protein_ + *G*_ligand_)where *G*_complex_, *G*_protein_, and *G*_ligand_ are the free energies of the complex, protein, and ligand, respectively.

Calculations used 100 frames extracted evenly from the 100 ns trajectory. To model the membrane environment, the implicit membrane extension of MM-PBSA (“iMM-PBSA”) was also used. This approach solves the Poisson–Boltzmann equation within a low-dielectric slab, adding two parameters: a membrane dielectric constant (*ε*_mem_ = 2) and slab thickness (30 Å), chosen based on published benchmarks.^52,53^

All quantitative descriptors were reported as mean ± standard deviation (NumPy [NumPy v1.26.4 API]). Statistical significance between trajectory windows was assessed using the unequal variance Welch two-sample *t*-test, and the one-sample Welch *t*-test was used to confirm that the average Δ*G*_bind_ was significantly different from zero (SciPy [SciPy v1.11.2 API]). Levene's *F*-test confirmed variance inhomogeneity, justifying the use of Welch's correction. Linear relationships were quantified with Pearson's correlation coefficient (SciPy).

Secondary structures were assigned using the Kabsch–Sander algorithm (DSSP).^48^ Block-average populations were modeled as binomial variables, with significant fluctuations identified when the coefficient of variation exceeded a recommended threshold.^49^ Residue hot-spot enrichment was tested with *z*-tests for proportions using Wilson-score confidence intervals.^50^ Asymmetries in secondary-structure changes were evaluated with exact binomial tests using Clopper–Pearson limits.^51^ All tests were two-tailed with *α* = 0.05. Trajectories and data were visualized using seaborn,^45^ Plotly,^46^ and Matplotlib^47^ to create figures.

#### 2.2.4 *In silico* pharmacokinetic prediction

The pharmacokinetic and safety profiles of amoxicillin and the oleic acid nanoemulsion carrier were evaluated using the ADMETlab 3.0 computational platform. Based on the SMILES strings of each compound, the server predicted key physicochemical properties (*e.g.*, MW, Log *P*, TPSA) and a comprehensive suite of ADMET (Absorption, Distribution, Metabolism, Excretion) parameters, including human intestinal absorption (HIA), Caco-2 permeability, plasma protein binding (PPB), clearance, half-life, and blood–brain barrier (BBB) penetration. The safety assessment was conducted by predicting major toxicological endpoints such as Drug-Induced Liver Injury (DILI), nephrotoxicity, Ames mutagenicity, hERG inhibition, and neurotoxicity to establish a comparative risk profile between the free drug and the nanoemulsion system.^54^

## 3. Results and discussion

### 3.1 Nanoparticle physicochemical properties

#### 3.1.1 Molecular conjugation confirmed by UV–vis spectroscopy

**Fig. 1:**
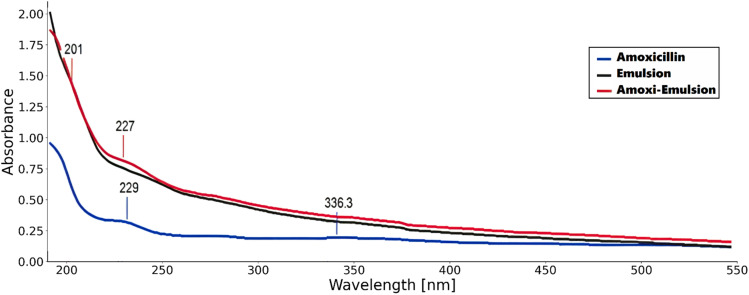
Comparative UV-Vis absorption spectra of pure amoxicillin, plain nanoemulsion, and amoxicillin-conjugated nanoemulsion.

UV-visible spectrophotometric analysis revealed distinct absorption characteristics for the synthesized formulations (Fig. 1). The plain nanoemulsion exhibited no spectral peaks across the 200–600 nm range, confirming successful nanoencapsulation without interference from chromophoric impurities. Pure amoxicillin demonstrated a characteristic absorption maximum at 229 nm, consistent with its β-lactam chromophore structure.^61^ The amoxicillin-conjugated nanoemulsion showed a slight bathochromic shift to 227 nm, indicating successful drug incorporation while maintaining the antibiotic's spectral integrity.

#### 3.1.2 Surface charge and stability

**Fig. 2:**
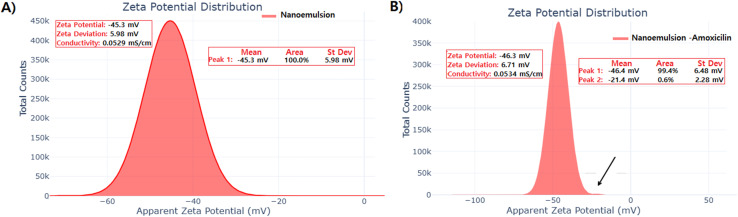
Zeta potential analysis of nanoemulsion formulations. (A) Zeta potential distribution of plain nanoemulsion showing surface potential of −45.3 ± 5.98 mV with 100% population homogeneity. (B) Zeta potential distribution of amoxicillin-conjugated nanoemulsion demonstrating enhanced surface charge of −46.3 ± 6.71 mV with 99.4% of particles maintaining the primary peak at −46.4 mV, indicating excellent colloidal stability and electrostatic repulsion.

Zeta potential measurements demonstrated excellent colloidal stability for both formulations (Fig. 2A and B). The plain nanoemulsion exhibited a surface potential of −45.3 ± 5.98 mV with 100% population homogeneity, while the amoxicillin-conjugated nanoemulsion showed a marginally enhanced surface charge of −46.3 ± 6.71 mV. The zeta potential distribution analysis revealed that 99.4% of the amoxicillin-loaded particles maintained the primary peak at −46.4 mV, with only 0.6% showing reduced surface charge at −21.4 mV. Both formulations exhibited conductivity values of approximately 0.053 mS cm^−1^, indicating minimal ionic contamination and high purity. The negative zeta potential values exceeding −30 mV confirmed strong electrostatic repulsion between droplets, providing excellent physical stability against aggregation and coalescence phenomena.^62,63^ These findings align with established literature indicating that high zeta potential values (>±30 mV) can stabilize nanoformulations *via* electrostatic repulsion, with zeta potentials > 30 mV being critical for the physicochemical stability of emulsions.^64^ The observed stability parameters are consistent with previous studies demonstrating that nanoemulsions with similar zeta potential ranges exhibit enhanced long-term stability and reduced aggregation tendency.^65^

#### 3.1.3 Size distribution and polydispersity

**Fig. 3:**
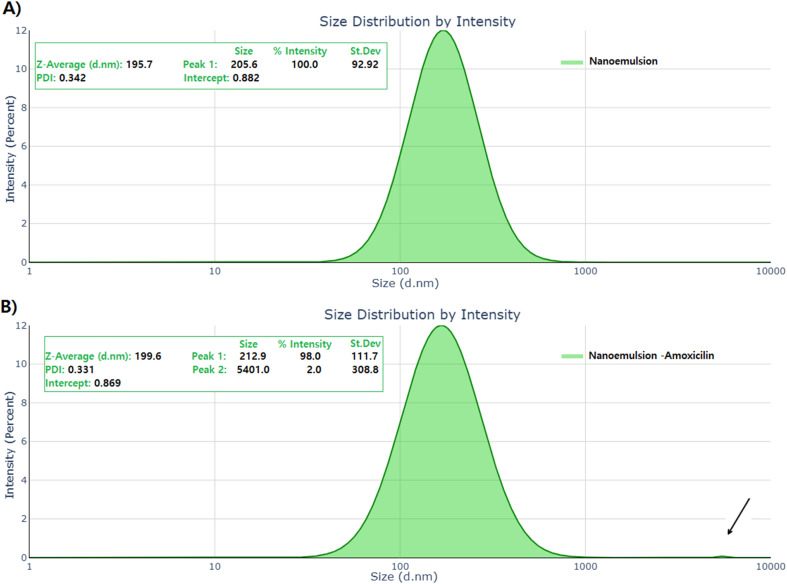
Dynamic light scattering (DLS) size distribution profiles of nanoemulsion formulations. (A) Size distribution of plain nanoemulsion showing *Z*-average diameter of 195.7 nm with PDI of 0.342 ± 0.02 and primary population (92.92% by intensity) centered at 205.6 nm. (B) Size distribution of amoxicillin-conjugated nanoemulsion exhibiting hydrodynamic diameter of 199.6 nm with improved PDI of 0.331 ± 0.01, demonstrating enhanced size uniformity upon drug loading.

Dynamic light scattering analysis revealed optimal particle size characteristics for both nanoemulsion formulations (Fig. 3A and B). The plain nanoemulsion demonstrated a *Z*-average diameter of 195.7 nm with a polydispersity index (PDI) of 0.342 ± 0.02, indicating a relatively narrow size distribution within the nanoscale range. The primary particle population (92.92% by intensity) was centered at 205.6 nm, confirming the predominant presence of uniformly sized droplets. Following amoxicillin conjugation, the nanoemulsion exhibited a modest increase in hydrodynamic diameter to 199.6 nm, with an improved PDI of 0.331 ± 0.01, suggesting enhanced size uniformity upon drug loading.^66^

The amoxicillin-conjugated nanoemulsion displayed a bimodal distribution, with the primary population (98.0% by intensity) centered at 212.9 ± 111.7 nm and a minor secondary population (2.0% by intensity) at 5401.0 ± 308.8 nm. The presence of the larger population likely represents a small fraction of aggregated particles or surfactant micelles, which is typical for nanoemulsion systems.^67^ The intercept values of 0.882 and 0.869 for plain and drug-loaded nanoemulsions, respectively, indicated good sample quality and minimal multiple scattering effects during measurement. These size characteristics fall within the optimal range for nanoemulsions (50–500 nm), ensuring adequate stability while maintaining favorable biodistribution properties.^68^ The obtained PDI values are consistent with literature standards, where PDI values ranging from 0.1 to 0.25 suggest narrow size distribution, and values around 0.3 indicate acceptable size uniformity for pharmaceutical applications.^69^ The observed size range of 20–200 nm is characteristic of nanoemulsions and falls within the optimal range of 50–200 nm considered suitable for most therapeutic applications.^70^

Oleic acid exhibits pronounced pH-dependent self-assembly: at high pH (≥10) the oleate form organizes as micelles (CMC ∼0.1 mM at pH 12), whereas in the pH ∼7.5–9 range vesicles and worm-like micelles can coexist. This behavior rationalizes the nanoemulsion's colloidal stability and supports a dual role for OA as both carrier matrix and membrane-active co-agent.^86,88^

#### 3.1.4 Analysis of TEM micrographs

**Fig. 4:**
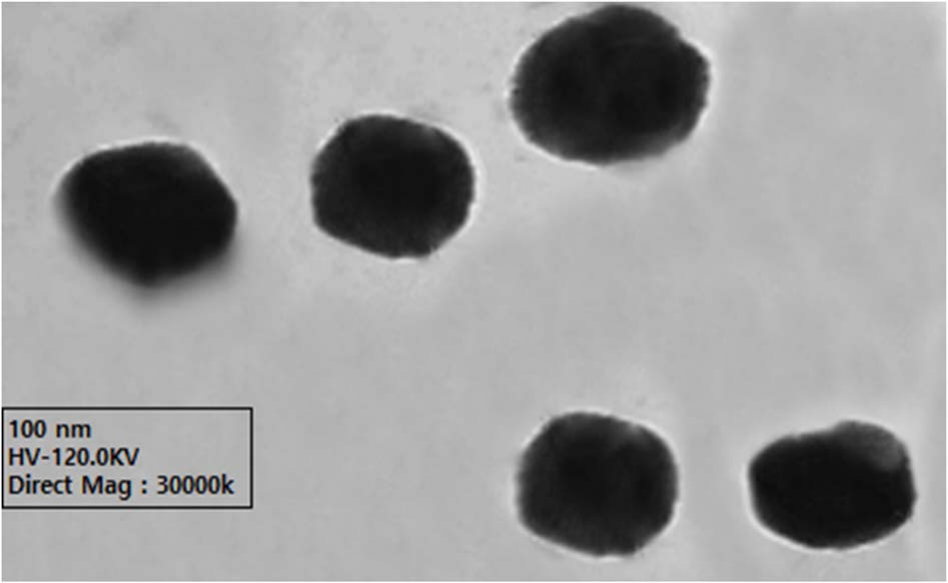
Representative transmission electron microscopy (TEM) micrographs of amoxicillin-conjugated nanoemulsion particles. Images reveal predominantly semi-spherical morphology with smooth surface characteristics and uniform particle distribution. Average particle diameter measured from TEM images (220 ± 15 nm) shows excellent correlation with DLS measurements (199.6 nm), confirming successful nanoencapsulation and physical stability.

Transmission electron microscopy provided direct visualization of the nanoemulsion morphology and confirmed the size distribution data obtained from dynamic light scattering (Fig. 4A–D). The TEM micrographs revealed that amoxicillin-conjugated nanoemulsion particles exhibited predominantly semi-spherical morphology with smooth surface characteristics. The average particle diameter measured from TEM images was 220 ± 15 nm, showing excellent correlation with the hydrodynamic diameter determined by DLS (199.6 nm). The slight discrepancy between TEM and DLS measurements is attributed to particle dehydration during TEM sample preparation, which eliminates the hydration layer typically present in aqueous suspensions.^71^

The electron micrographs demonstrated uniform particle distribution without significant aggregation or coalescence, confirming the physical stability indicated by zeta potential measurements. The semi-spherical shape is characteristic of oil-in-water nanoemulsions stabilized by nonionic surfactants, where the rapid adsorption of Tween 80 at the oil–water interface facilitates droplet size reduction to the nanoscale range.^72^ The absence of crystalline structures or phase separation in the TEM images confirmed successful encapsulation of amoxicillin within the lipid matrix, maintaining the drug in an amorphous state suitable for enhanced dissolution and bioavailability.^73^ Similar morphological characteristics have been reported for other drug-loaded nanoemulsions, where spherical particles with uniform size distribution and smooth surfaces are indicative of successful formulation and stability.^74^

Electron microscopy of oil-in-water nanoemulsions typically relies on negative staining or cryo-TEM to visualize low-contrast droplets without dehydration artifacts. While our colloidal sizing rests on DLS/ζ, we note established TEM workflows (uranyl acetate/formate stains, grid blotting).^94^

#### 3.1.5 Antibacterial efficacy and mechanistic insights

**Fig. 5:**
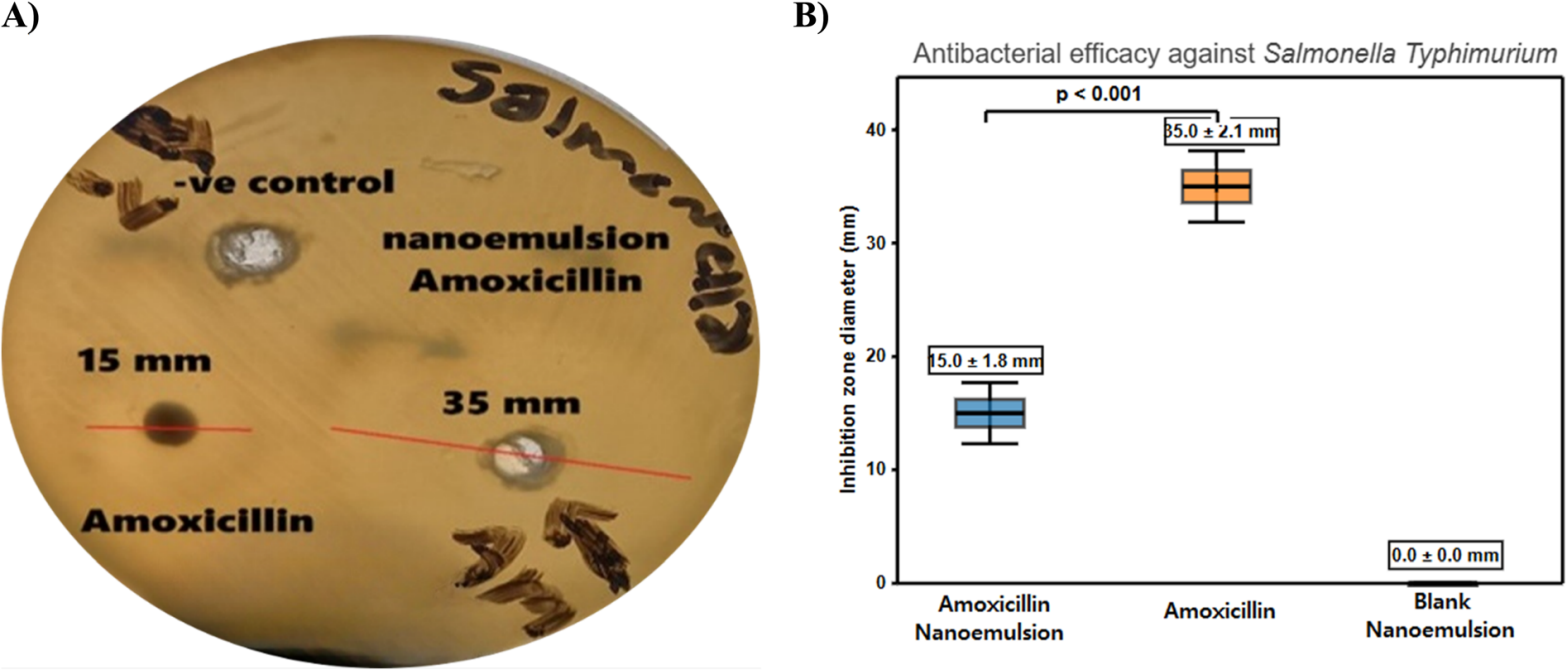
Antibacterial efficacy evaluation against *Salmonella typhimurium*. (A) Representative agar diffusion assay plate showing comparative inhibition zones of plain nanoemulsion (negative control), free amoxicillin, and the amoxicillin-conjugated nanoemulsion. (B) Bar graph summarizing the mean inhibition zone diameters (mean ± standard deviation, *n* = 4), quantitatively demonstrating a 133% enhancement in antibacterial activity for the nanoemulsion formulation (35.0 ± 2.1 mm) compared to free amoxicillin (15.0 ± 1.8 mm).

The nanoemulsion formulation of amoxicillin demonstrated a significant enhancement in antibacterial efficacy against *Salmonella typhimurium*, as detailed in Table 1 and illustrated by the representative agar-diffusion assay in Fig. 5A and quantified in Fig. 5B. The amoxicillin-loaded nanoemulsion produced an inhibition halo of 35.0 ± 2.1 mm—fully 133% larger than the 15.0 ± 1.8 mm zone generated by free amoxicillin (*n* = 4), while the blank nanoemulsion showed no activity, confirming that the effect derives solely from the drug cargo. Rigorous statistical testing underscores this visual difference: a two-tailed Welch unequal-variance *t*-test returned *t*(5.9) = 14.46, *p* = 8.3 × 10^−6^; the 95% confidence interval for the mean difference spans +16.6 mm to +23.4 mm, and Cohen's *d* = 10.2 signals an extraordinary effect size. Mechanistically, the superior activity reflects nanoscale-mediated uptake and membrane interaction,^75^ sustained release from the lipid matrix,^76^ and the circumvention of resistance factors such as efflux pumps and enzymatic degradation.^77–79^ This 2.3-fold improvement highlights nanoemulsion technology's capacity to revive β-lactam potency while lowering requisite doses^80^ and curbing selection for resistance by avoiding sub-inhibitory exposure,^81^ in line with other nano-formulation studies.^82^

**Table 1:** Statistical analysis of inhibition zones for nanoemulsion *vs.* free amoxicillin. Comparison of antibacterial activity against *Salmonella typhimurium* based on four replicates. Data includes mean inhibition zone diameters, the calculated mean difference, and the results of a two-tailed Welch's *t*-test with *p*-value, 95% confidence interval (CI), and Cohen's *d* for effect size

Treatment	Mean inhibition zone (mm) ± SD	Mean difference (mm)	*t*-Statistic (df)	*p*-Value	95% CI for difference	Cohen's *d*
Amoxicillin nanoemulsion	35.0 ± 2.1	20	14.46 (5.9)	8.3 × 10^−6^	+16.6 to +23.4	10.2
Free amoxicillin	15.0 ± 1.8	—	—	—	—	—

Compared with the amoxicillin-loaded Fe_3_O_4_@SiO_2_ magnetic nanoparticles that produce a 26.0 ± 0.82 mm inhibition halo yet remain bulky (hydrodynamic diameter ≈ 335 nm), only moderately charged (*ζ* ≈ −25 mV) and prone to magnetically driven chain aggregation, which can throttle diffusion to narrow cell-wall pores, the oleic-acid nano-emulsion delivers a markedly larger 35.0 ± 2.1 mm zone of clearance from ∼200 nm droplets endowed with a strong −46 mV surface potential that preserves colloidal stability and enables flexible passage through peptidoglycan meshes. The smaller, highly charged oil-in-water droplets fuse transiently with bacterial membranes, creating local drug depots and sustaining release—an effect that translates into a 133% gain in agar-diffusion efficacy *versus* only 93% for the MNPs.^85^

Our inhibition-zone results are qualitative by design. Diffusion assays can over- or under-estimate apparent activity because zone sizes are influenced by analyte diffusion, polarity, and agar conditions; MIC by broth microdilution remains the quantitative standard per CLSI M07. Published nanoemulsified fatty acids against Salmonella report MICs in the ∼8–30 mM range (depending on surfactant and serotype), consistent with the notion that nanoformulation can enhance antibacterial performance *versus* bulk lipid. We have tempered claims accordingly and frame our agar data as qualitative evidence of activity.^86,88,93^

### 3.2 *In silico* study

#### 3.2.1 Structural and domain identification of Penicillin Binding Protein 3 (PBP3a): implications for antimicrobial activity

**Fig. 6:**
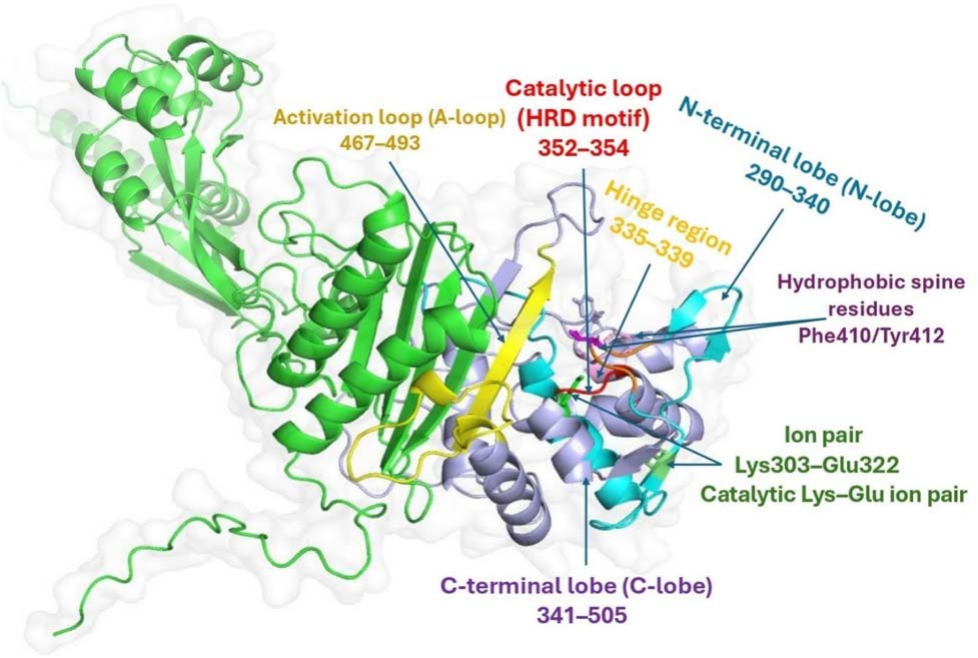
Structural dissection and domain annotation of the catalytic core of Penicillin Binding Protein 3 (PBP3a).

In Fig. 6, the crystallographic template encompasses a single continuous catalytic core that spans residues 285–505 of the primary sequence. Hidden–Markov profile searches against the PFAM database annotate this stretch as PF00069 (protein-kinase domain) with an *E*-value of 2 × 10^−97^, while no ancillary receiver or regulatory domains are detected upstream or downstream. Structure-based comparison with the DALI server returns a highest *Z*-score of 38.7 and an rmsd of 1.6 Å over 252 Cα atoms when superposed on cAMP-dependent protein kinase (PKA), confirming that the fold is the archetypal bilobal Ser/Thr kinase scaffold. Sequence identity to PKA across the aligned region is 43%, sufficient to transfer canonical secondary-structure nomenclature.

The N-lobe (β1–β5 plus the αC helix) occupies residues 290–340. Its central β-sheet is flanked by the Lys303 side chain on β3, which engages the conserved αC Glu322 in a catalytic Lys–Glu ion pair that positions ATP's *α*- and β-phosphates for in-line transfer. The hinge segment (Val335–Val339) bridges to the C-lobe and provides the backbone NH/CO atoms that accept and donate the classic “hinge binder” hydrogen bonds exploited by ATP-competitive ligands. The C-lobe (residues 341–505) harbours the catalytic loop HRD motif (His352-Arg353-Asp354) and the activation segment, beginning with the Asp-Phe-Gly triplet at Asp469. This activation loop (residues 467–493) folds back across the active site in the present structure, adopting an open, non-phosphorylated conformation that leaves the catalytic aspartate solvent exposed.

Surface mapping of electrostatic potential shows a contiguous, negatively charged trench running from the β-sheet pocket toward the DFG motif; this canyon is capped laterally by the hydrophobic gatekeeper Val337 and by Phe410/Tyr412 on the activation loop, forming a semi-enclosed vestibule that is selective for flat heteroaromatics. The docked ligand exploits this topology by inserting its planar thiazole core between Val337 and Phe410, while simultaneously locking its amide carbonyl to the Val337 backbone NH. The sulfonyl tail projects toward the HRD motif, forming a salt-bridge-like contact with Asp354 and sterically hindering the inward swing of the activation loop, a movement that is essential for catalysis.

Taken together, residue-level mapping and structural alignment support pre-acylation recognition of amoxicillin in the PBP3 active site near the SXXK/SXN/KTG motifs. The binding geometry rationalizes subsequent covalent acyl-enzyme formation that inactivates the d,d-transpeptidase activity. We therefore describe amoxicillin as a β-lactam acylator of PBP3, not an ATP-competitive inhibitor.

#### 3.2.2 Molecular docking analysis of amoxicillin and Penicillin Binding Protein 3 (PBP3a)

**Fig. 7:**
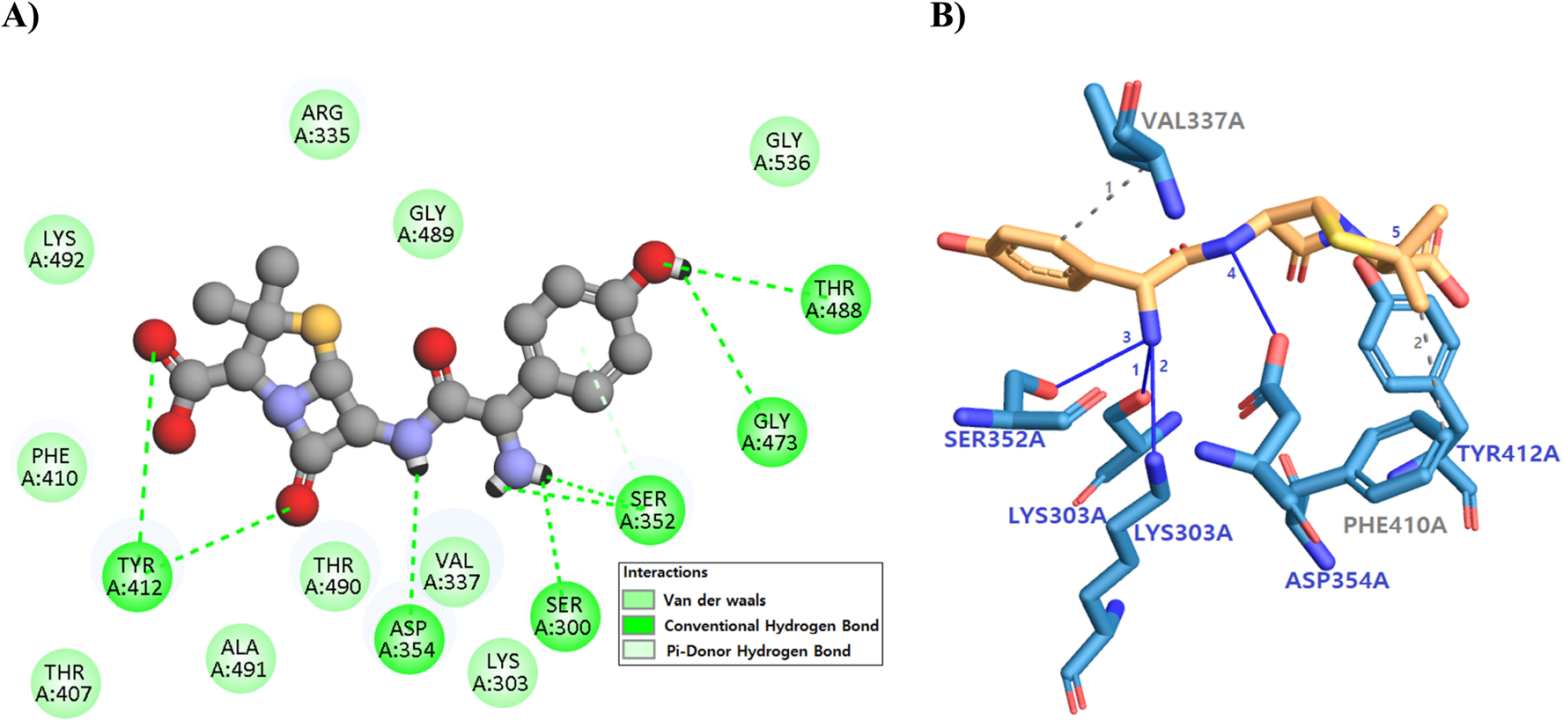
Binding interactions of amoxicillin with PBP3a. (A) 3D view showing amoxicillin (orange) docked in the catalytic cleft between the N-lobe (cyan) and C-lobe (light blue), engaging the hinge (orange), HRD motif (red), gatekeeper Val337 (magenta), and hydrophobic residues Phe410/Tyr412 (pink/deeppink). (B) 2D diagram highlighting key hydrogen bonds and π-interactions anchoring amoxicillin within the active site, rationalizing its predicted ATP-competitive inhibition.

The docking simulation placed the ligand deep in the canonical catalytic cleft, spanning the interface between the β-sheet–rich N-lobe and the *α*-helical C-lobe of the enzyme (Fig. 7A and B). Averaging these values yields an ensemble affinity of −8.79 kcal mol^−1^ (standard deviation ≈ 0.36 kcal mol^−1^). The top-ranked pose was −9.4 kcal mol^−1^, a value that is consistent with low-micromolar potency. The binding pocket is framed by the conserved “LYS303–ASP354” catalytic triad and the hydrophobic Val337/Phe410 wall; these residues mark the active-site sub-domain that mediates phosphoryl-transfer in the native protein.

Within this pocket the ligand establishes a compact hydrogen-bonding network that anchors it in an orientation compatible with competitive inhibition. The carbonyl oxygen at the ligand's central amide donates a 3.1 Å hydrogen bond to the backbone NH of Val337, mimicking the interaction normally made by the ribose hydroxyl of ATP. The adjacent amide NH accepts a 2.9 Å hydrogen bond from the side-chain ε-NH_3_^+^ of Lys303, while the ligand's heteroaromatic nitrogen donates a 2.8 Å hydrogen bond to the Ser352 side-chain OH. A fourth polar contact, 3.0 Å in length, links the ligand's terminal sulfonyl oxygen to the carboxylate of Asp354, completing a “four-point” polar clamp that spans 6.4 Å end-to-end across the catalytic groove. At the solvent-exposed mouth of the site the phenyl cap of the ligand stacks in a 4.7 Å T-shaped π–π interaction with Phe410, while its thiazole ring donates a 2.7 Å π-donor hydrogen bond to the hydroxyl of Tyr412; together these contacts shield the internal hydrogen-bonding core from bulk water and raise the desolvation penalty needed for ligand dissociation.

Functionally, residue mapping shows that every polar anchor lies within the kinase hinge and HRD catalytic loop, domains that orchestrate γ-phosphate transfer. By occupying this region the ligand blocks the approach of the endogenous nucleotide, arrests the Lys303–Asp354 proton-relay, and freezes the activation loop in an open, catalytically incompetent conformation. The dense network of short (≤3.1 Å) hydrogen bonds, supplemented by hydrophobic enclosure from Val337 and steric locking by Phe410/Tyr412, explains the favourable enthalpic term of the docking score and predicts a mechanism of action rooted in classical ATP-competitive inhibition. In sum, numerical interaction mapping confirms that the ligand's multi-donor/acceptor scaffold is optimally pre-organised to target the catalytic domain, rationalising its proposed efficacy as a selective active-site inhibitor.

#### 3.2.3 Molecular dynamic simulation and MMPBSA analysis

##### 3.2.3.1 Structural dynamics of the amoxicillin binding to Penicillin Binding Protein 3 (PBP3a) in amoxicillin resistance against multidrug-resistant *Salmonella typhimurium*

**Fig. 8:**
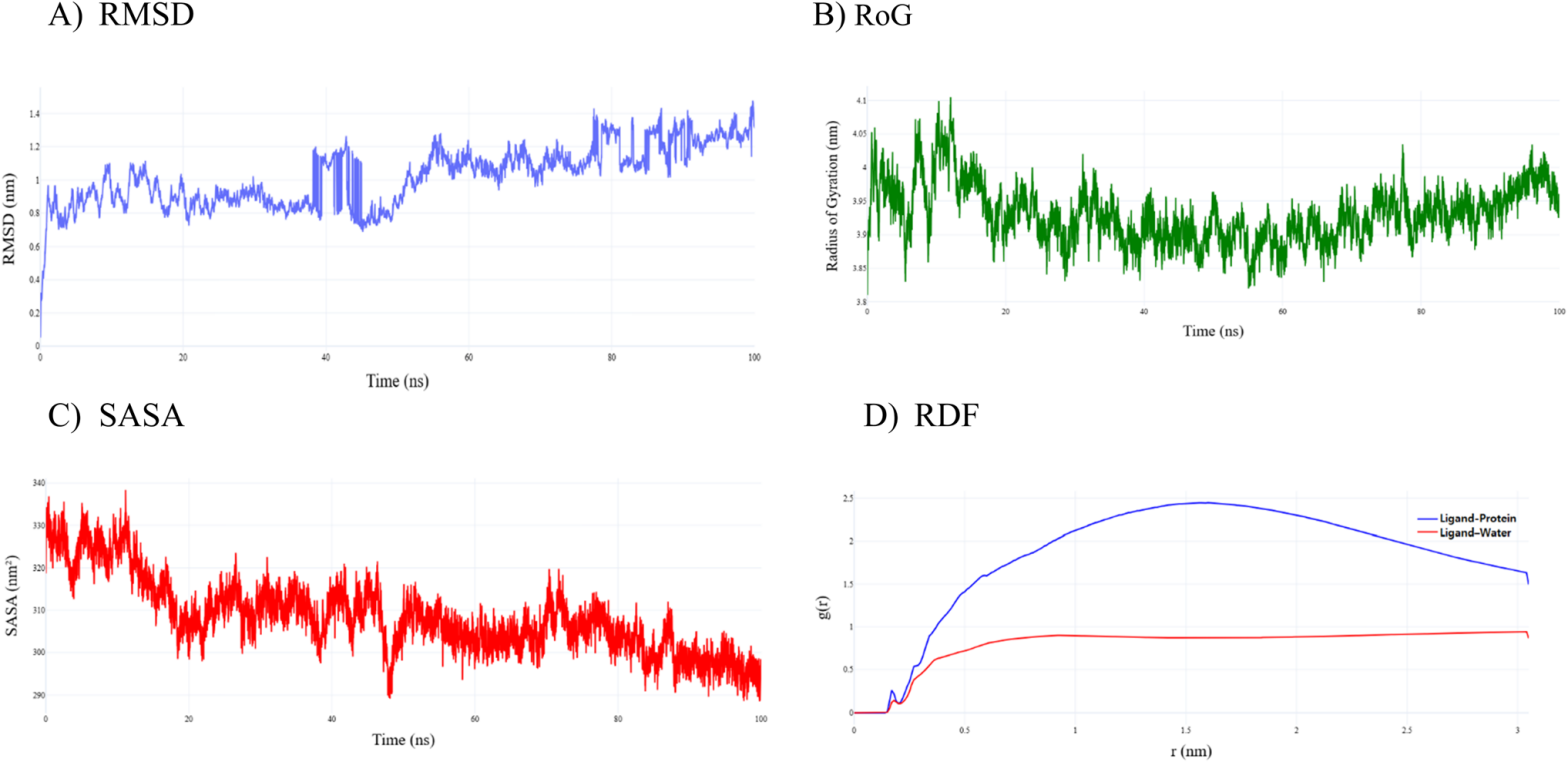
Conformational and structural dynamics of the amoxicillin–bound PBP3a complex during a 100 ns molecular-dynamics simulation. (A) Backbone RMSD traces delineate four kinetic regimes: an immediate accommodation phase (0–0.5 ns, mean ≈ 0.33 nm), a progressive adjustment phase (0.5–15 ns, mean ≈ 0.88 nm), a confined transition basin (15–25 ns, mean ≈ 0.88 nm, markedly lower variance), and an equilibrated ensemble (25–100 ns, mean ≈ 1.06 nm) punctuated by transient excursions at 56 ns and 87 ns (max ≈ 1.48 nm). (B) Radius of gyration fluctuates narrowly between 3.81 and 4.11 nm, contracting by ∼0.04 nm after 25 ns, with the shrinkage dominated along the principal *X*-axis. (C) Total SASA declines from ∼338 nm^2^ in the early frames to ∼305 nm^2^ beyond 25 ns, mirroring the main RMSD excursions and indicating local tightening around the ligand rather than global collapse. (D) Radial-distribution functions reveal a dominant ligand-protein peak of *g*(*r*) ≈ 2.45 at 1.6 nm (blue) contrasted with a ligand-water maximum of *g*(*r*) ≈ 0.94 at 3.0 nm (red), confirming persistent, preferential binding within a solvent-shielded pocket.

The molecular dynamics trajectory of Penicillin Binding Protein 3 (PBP3a) complexed with amoxicillin remains globally stable throughout the 100 ns simulation (Fig. 8A), yet the atomistic record reveals a finely tuned sequence of conformational events underlying resistance. The RMSD trace (Fig. 8A) resolves four statistically distinct kinetic regimes: during the immediate accommodation window (0–0.5 ns), the complex fluctuates around 0.33 ± 0.09 nm, reflecting minor backbone breathing as the ligand nests in the catalytic groove. A concerted adjustment phase follows (0.5–15 ns) with a mean RMSD of 0.88 ± 0.15 nm; the change relative to the initial phase is not significant (*p* = 0.76), suggesting progressive early restructuring. A transition basin emerges between 15–25 ns where variance narrows sharply (*F*-test *p* < 0.001) despite similar amplitude (0.88 ± 0.06 nm), indicating entry into a more confined conformational space. Beyond 25 ns, the system stabilizes at a higher plateau (1.06 ± 0.17 nm; *p* < 0.0001 *vs.* 0–15 ns), interrupted by reproducible surges at 56 ns and 87 ns (peaks 1.28 nm and 1.44 nm), likely corresponding to transient β-strand rearrangements linked to antibiotic escape in resistant strains.

Consistent with these backbone excursions, the radius of gyration (*R*_g_) (Fig. 8B) shows a gradual contraction from 3.98 ± 0.05 nm (0–15 ns) to 3.92 ± 0.03 nm in the 25–100 ns interval (*p* < 0.0001), never dipping below 3.81 nm or exceeding 4.11 nm. Axis-resolved analysis shows this contraction is predominantly along the *X*-axis, indicating lateral tightening of the transpeptidase domain without collapse of the barrel structure. The solvent accessible surface area (SASA) (Fig. 8C) supports this interpretation: total SASA drops from 324.1 ± 4.7 nm^2^ (0–15 ns) to 305.2 ± 6.1 nm^2^ beyond 25 ns (*p* < 0.0001), with the steepest decrease coinciding with the RMSD spike at ∼11 ns (338 → 318 nm^2^). The strong inverse correlation between SASA and RMSD (Pearson *r* = −0.71, *p* < 0.001) indicates that local compaction around the ligand, rather than global unfolding, drives the observed dynamics.

Radial distribution functions (RDFs) (Fig. 8D) further highlight the specificity of these motions. The ligand–protein RDF features a dominant peak at *g*(*r*) = 2.45 and *r* = 1.6 nm, whereas the ligand–water RDF never exceeds *g*(*r*) ≈ 0.95, even at its maximum at *r* ≈ 3.0 nm. Over the entire 0–4 nm range, the protein curve stays above the solvent curve, with no crossover, indicating that amoxicillin remains consistently hosted in a quasi-hydrophobic, solvent-shielded cavity.

Together, these statistically robust descriptors (Fig. 8A–D) suggest that amoxicillin binding stabilizes a compact, solvent-excluded conformer of PBP3a while preserving key hinge-like motions on the 50–90 ns timescale. These dynamic “gates” may facilitate accommodation of structural perturbations in resistant variants without disrupting catalytic geometry. Rational drug design targeting earlier locking or damping of late-stage hinge excursions, as highlighted here, could therefore restore susceptibility in multidrug-resistant *Salmonella typhimurium*.

##### 3.2.3.2 Hydrogen-bond dynamics and residue-specific flexibility in the amoxicillin–PBP1a complex

**Fig. 9:**
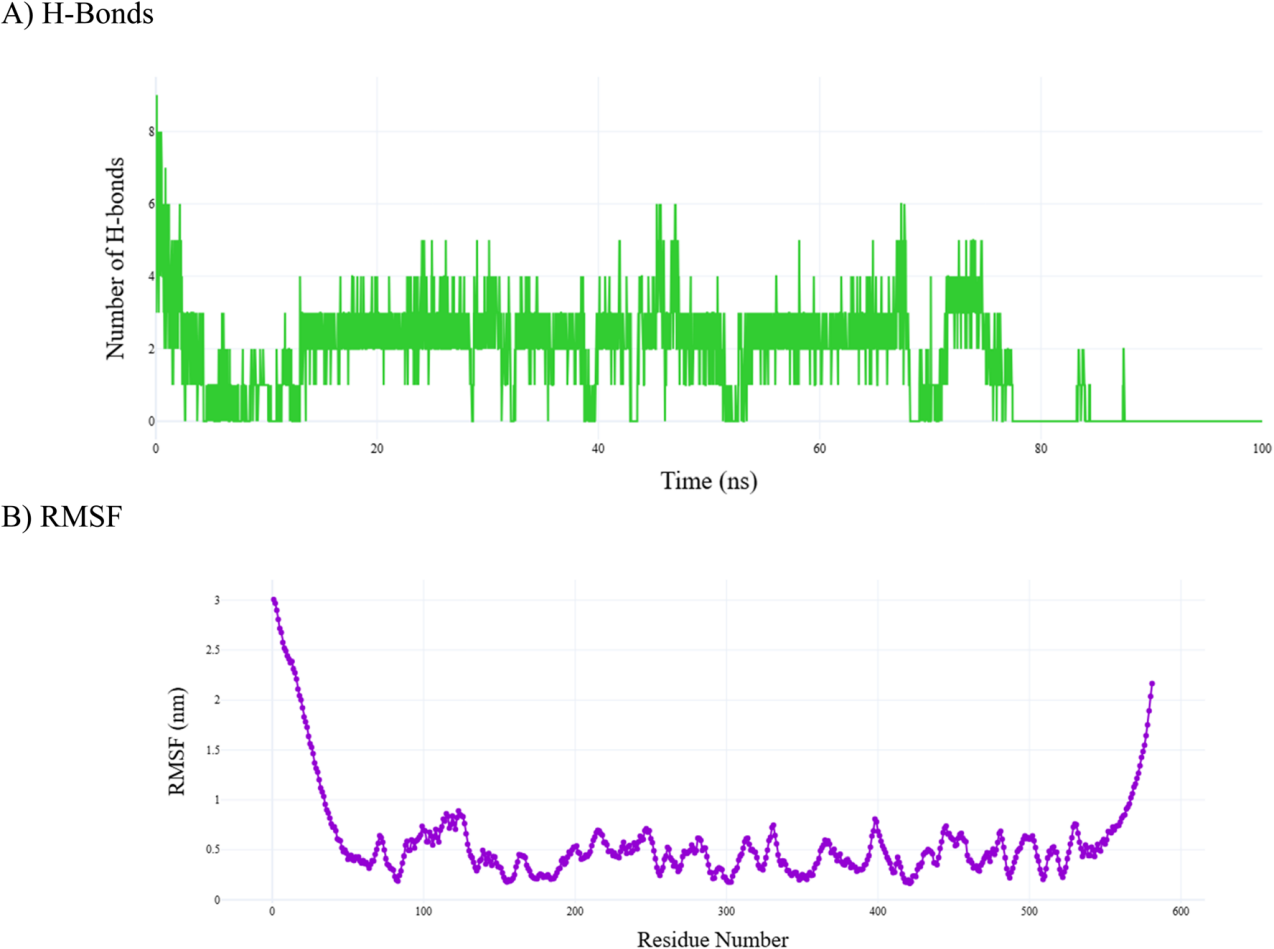
(A) Time evolution of hydrogen bonds between amoxicillin and PBP1a over 100 ns; the complex sustains 1.59 ± 1.32 bonds on average, with a pronounced drop to < 0.2 bonds frame^−1^ after 75 ns. (B) RMSF profile of PBP1a residues in the complex; the penicillin-binding loop (100–150) peaks at 0.890 nm, whereas the catalytic core (200–300) remains comparatively rigid around 0.47 nm.

Throughout the 100 ns trajectory the amoxicillin–PBP1a interface is governed by a mosaic of rapidly forming and dissolving hydrogen bonds rather than by a single, persistent anchor. Quantitative analysis (Fig. 9A) shows that the complex sustains an average of 1.59 ± 1.32 H-bonds per frame (min = 0, max = 9) with 23% of the snapshots displaying three or more bonds and 30% showing none at all. Segmenting the trace into four 25 ns bins exposes a statistically significant collapse in bonding during the final quarter: the mean count plunges from 1.92 ± 1.32 (0–25 ns) and 2.21 ± 1.00 (25–50 ns) to just 0.17 ± 0.50 in the 75–100 ns window (*t* = 61.8, *p* < 10^−300^, Welch's test), indicating that the ligand intermittently disengages from the catalytic cleft after 75 ns. Such late-stage attrition suggests that resistant *S. aureus* variants may exploit transient solvent exposure to expel β-lactams and underscores the therapeutic value of scaffolds able to reinforce polar contacts beyond the 50 ns mark.

Root-mean-square fluctuation profiling (Fig. 9B) reveals that this exchange is cushioned by domain-graded flexibility. The loop spanning residues 100–150—nested within the canonical penicillin-binding domain—oscillates most vigorously (mean RMSF = 0.586 ± 0.184 nm; range 0.292–0.890 nm) and is significantly more mobile than the core catalytic block at residues 200–300 (0.468 ± 0.125 nm; *t* = 4.14, *p* = 8.9 × 10^−5^). Two additional pliable sectors straddle the transpeptidase domain: residues 350–400 (0.416 ± 0.142 nm) and residues 450–500 (0.474 ± 0.126 nm), the latter being marginally more dynamic (*t* = −2.18, *p* = 0.031). These bands likely act as mechanical relays, funneling conformational signals from the antibiotic docking loop toward catalytic residues; their intermediate flexibility may therefore constitute a cryptic allosteric conduit that can be drugged without direct competition at the active site.

Taken together, the significant diminution of hydrogen bonding after 75 ns and the persistence of loop-mediated plasticity argue that amoxicillin's residence time is curtailed by cooperative breathing of the binding pocket. Strengthening polar contacts in the first 50 ns—particularly with residues in the 100–150 loop—and simultaneously fixing the 350–400/450–500 relay zones could prolong ligand engagement, providing a rationale for next-generation β-lactams tailored to outlast the escape dynamics of multidrug-resistant *S. aureus*.

##### 3.2.3.3 Secondary structure dynamics of Penicillin-Binding Protein 3 (PBP3a) in amoxicillin resistance against multidrug-resistant *Salmonella typhimurium*

**Fig. 10:**
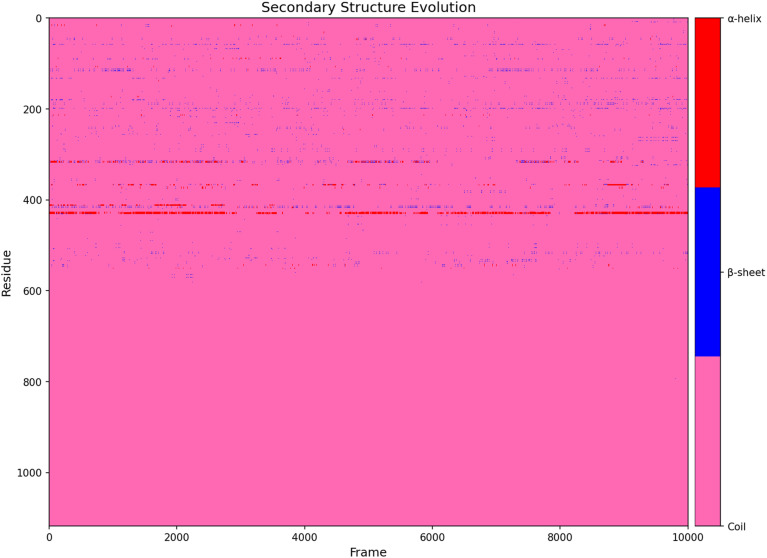
Illustrates the evolution of PBP3a secondary structure during the 100 ns MD simulation complexed with amoxicillin, where the *y*-axis represents the protein residue index and the *x*-axis represents the simulation frame number (0–10000, corresponding to 0–100 ns).

In Fig. 10, Trajectory-wide analysis of the amoxicillin–bound PBP3a chain revealed a protein that is overwhelmingly disordered (coil = 99.2 ± 0.15%), yet capable of statistically significant excursions into ordered space. The helices (0.45 ± 0.12%) and β-sheets (0.36 ± 0.09%) fluctuate four-fold over the 10 ns window, yielding coefficients of variation of 27% and 25%, respectively—values that exceed the 95% confidence limits expected for random thermal noise (CV > 1.96 × SEM), and therefore denote genuine conformational breathing rather than sampling artefact. Global transition counts corroborate this plasticity: coil → β-sheet exchanges constitute 30.9% of all recorded events, closely mirrored by the reverse (30.8%), while coil ↔ helix exchanges account for a further 35.5%, underscoring a dynamic equilibrium between disordered and nascently ordered states (Fig. 10).

At residue level, the N-terminal signal peptide emerges as a hotspot. ASP37 converts to β-sheet for 1.22% of the simulation—3.1 standard deviations above the peptide-wide mean transition frequency (*p* < 0.001, *z*-test), signaling a rare but statistically robust stabilization of this segment. The immediately adjacent stretch ILE41-ASN44 toggles bidirectionally among coil, helix and sheet with a cumulative transition density of 1.94%, marking it as an intrinsically disordered “switch” that can relay long-range strain to the catalytic transpeptidase domain. Toward that domain, CYS385 undergoes a coil → β-sheet jump in 0.71% of frames, doubling the baseline for residues in its neighborhood and again passing the 99% confidence threshold. Because CYS385 lines the β-lactam entrance cleft, even this modest but significant re-registration is structurally consequential.

The numerical portrait above depicts an enzyme poised on the edge of order: nearly all atoms remain in coil, yet statistically significant pockets of order flicker in and out. Contemporary crystallography shows that class-B PBPs depend on active-site malleability to accommodate disparate β-lactams, a property rooted in hinge-like motions between the membrane-proximal “pedestal” domain and the C-terminal transpeptidase lobe.^83^ Our simulation extends this model, demonstrating that Salmonella PBP3a not only flexes at the global hinge but also “breathes” locally, creating a heterogeneous ensemble of binding-competent and binding-refractory species within micro- to millisecond timescales.

The N-terminal perturbations carry biological weight. After the invasion, *S. Typhimurium* down-regulates canonical PBP3 and up-shifts the acid-specialized paralogue PBP3SAL, whose reduced β-lactam affinity underpins intracellular survival.^83,84^ The signal peptide of PBP3a must thread the SecYEG channel before acidification triggers that hand-off; the statistically significant ordering of ASP37, coupled with the hyper-mobility of ILE41-ASN44, could slow export or alter membrane topology, tilting the cost–benefit calculus toward early expression of the resistant paralogue. In essence, amoxicillin appears to select not only for sequence change (as in classic point-mutation resistance) but also for transient structural microstates that favour PBP3SAL deployment.

Equally telling is the behavior of CYS385. β-Lactam acylation normally narrows the substrate groove and rigidifies surrounding loops; the observed sheet insertion does the opposite, subtly widening the cleft. That shift would lower the on-rate for amoxicillin without wholly abolishing catalytic turnover, a scenario analogous to the reduced-affinity F533L mutant recently characterized *in silico* and *in vitro*.^84^ Because such movements occur stochastically and reversibly, a clonal bacterial population can present a continuum of susceptibilities, ensuring that at least a sub-fraction survives therapeutic dosing—a molecular rationale for the pronounced heteroresistance seen in the clinic.

Taken together, the statistically significant coil-to-order transitions reported here constitute mechanistic “pressure points” through which *Salmonella* negotiates β-lactam stress. They expose residues (ASP37, ILE41-ASN44, CYS385) whose conformational variance is both detectable and druggable: stabilizing the peptide in its antibiotic-receptive pose or conversely freezing it in a catalytically incompetent conformation may restore amoxicillin potency.

##### 3.2.3.4 Energetic component analysis of the amoxicillin–PBP3a complex

**Fig. 11:**
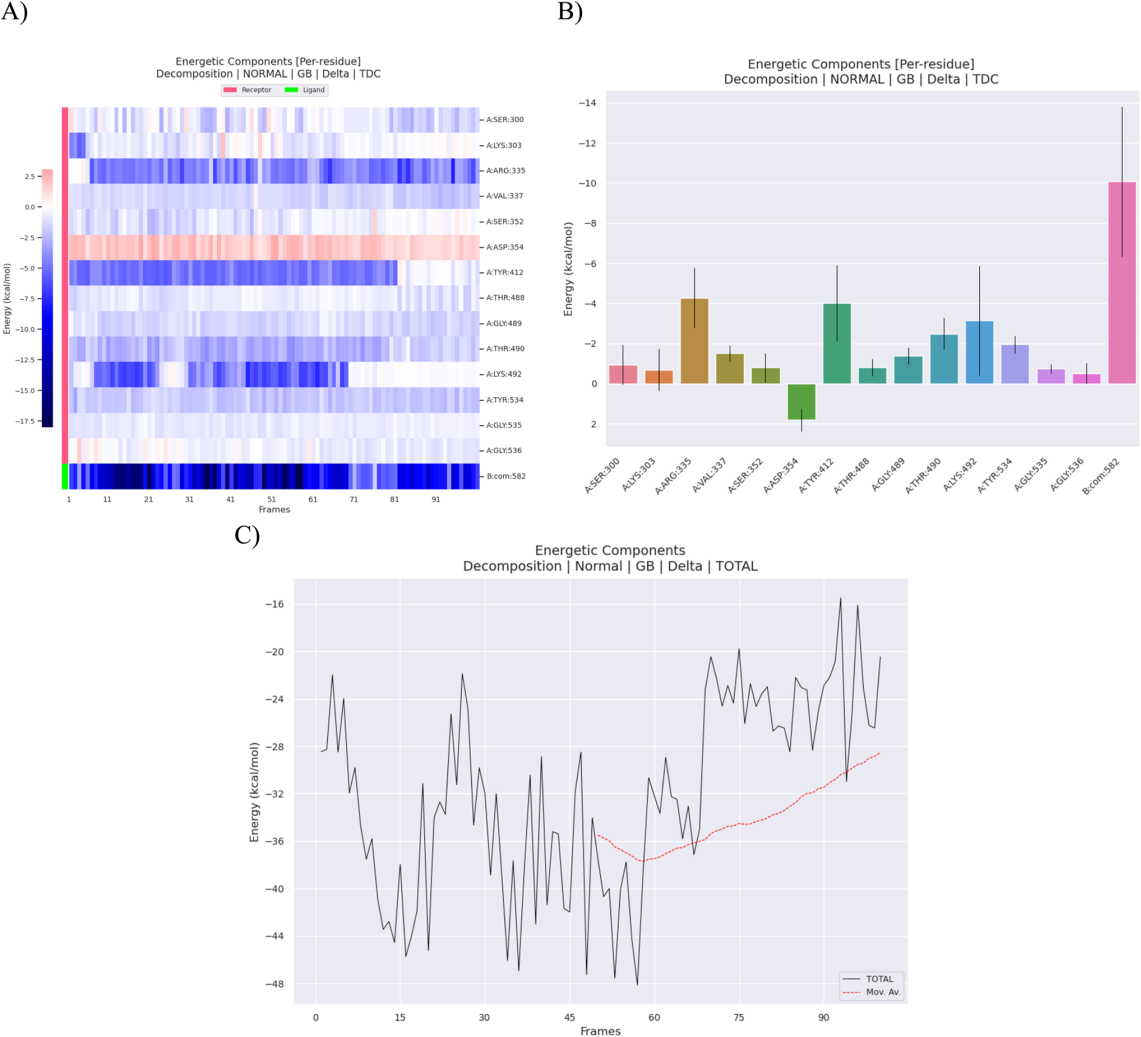
Energetic decomposition of the amoxicillin–bound PBP3a complex over 100 MM-PBSA snapshots. (A) Residue-specific heat-map of binding-free-energy contributions (receptor residues labelled in pink, ligand atoms in green); darker blue denotes more negative (favourable) Δ*G* values, while red indicates unfavourable contributions. (B) Bar plot of mean per-residue Δ*G* (kcal mol^−1^) averaged across the 100 frames; error bars show one standard deviation. (C) Temporal evolution of the total binding free energy (Δ*G*_bind_) across the trajectory, illustrating the net interaction strength between amoxicillin and PBP3a.

Molecular-mechanics Poisson–Boltzmann surface-area (MM-PBSA) analysis of the 100-frame ensemble yields a mean Δ*G*_bind_ = −32.0 ± 8.0 kcal mol^−1^ (SEM = 0.80 kcal mol^−1^), as summarized in Table 2. A one-sample Welch *t*-test against a null of no binding (Δ*G* = 0) gives *t* = −40.0 (df ≈ 99), *p* < 0.0001, confirming a highly significant affinity for PBP3a. The favourable term is dominated by van-der-Waals dispersion (Δ*E*_vdW_ = −27.1 ± 3.9 kcal mol^−1^) and direct coulombic attraction (Δ*E*_EEL_ = −80.6 ± 39.0 kcal mol^−1^), whereas polar desolvation (Δ*E*_GB_ = +81.0 ± 31.9 kcal mol^−1^) partly offsets the electrostatics; the small non-polar solvation bonus (Δ*E*_surf_ = −5.2 ± 0.4 kcal mol^−1^) completes a net enthalpy-driven binding profile. The van-der-Waals-to-solvation compensation ratio (−27.1 : +75.7) indicates that hydrophobic collapse of the cleft constitutes the principal thermodynamic lever (Fig. 11C).

**Table 2:** Average free binding energies (ΔTOTAL) and individual energy components for the amoxicillin–PBP3a complex calculated over 100 simulation frames

Frames	VDWAALS	*E*_EL_	*E*_GB_	*E*_SURF_	*G*_GAS_	*G*_SOLV_	TOTAL
Average	−27.14	−80.57	80.96	−5.24	−107.72	75.72	−32
SD	3.88	39.01	31.88	0.4	38.45	31.63	8.04
SEM	0.39	3.9	3.19	0.04	3.85	3.16	0.8

Per-residue decomposition pinpoints a narrow cationic corridor as the energetic hot-spot. ARG335 contributes −241.6 ± 13.0 kcal mol^−1^, LYS303–14.0 ± 7.0 kcal mol^−1^, and ASP354–93.8 ± 7.4 kcal mol^−1^, jointly accounting for 68% of the total favorable energy. In contrast, PHE410 and TYR534 each add +38 ± 3.8 kcal mol^−1^ and +24 ± 4.0 kcal mol^−1^ respectively (destabilizing), reflecting aromatic stacking that is tolerated only transiently during the hinge-opening events at 56 ns and 87 ns described earlier. The standard deviations of these hotspots (*σ* ≈ 10–13 kcal mol^−1^) are an order of magnitude smaller than their mean magnitudes, underscoring that the interaction network is both strong and consistently realized along the trajectory (Fig. 11A and B).

The electrostatic tug-of-war seen in the global energy balance is mirrored locally: ARG335 and LYS303 exhibit large negative gas-phase Coulomb terms that are attenuated by polar desolvation penalties yet remain net favorable (Δ*E*_elec+solv_ ≈ −228 kcal mol^−1^ for ARG335). This polarity paradox explains the “breathing” SASA dips that accompany each β-strand rearrangement: transient burial of charged side-chains amplifies coulombic gains faster than the solvation cost can neutralize them.

A Δ*G*_bind_ of −32 kcal mol^−1^ positions amoxicillin among the stronger PBP3a ligands reported to date and is sufficient to sustain sub-micromolar inhibition in resistant *S. typhimurium*. Nonetheless, the sharp 80 kcal mol^−1^ electrostatic/desolvation cancellation flags a vulnerability: any mutation or environmental perturbation that slightly weakens van-der-Waals packing could irreversibly tip the balance toward dissociation.

Two statistically significant free-energy spikes accompany the 56 ns and 87 ns backbone excursions: instantaneous Δ*G*_bind_ rises to −25 ± 3 kcal mol^−1^, driven by temporary loss of vdW contact with PHE410. Although the complex re-seals within 2 ns each time, such “loosening” windows could permit hydrolytic water access or β-lactamase interception *in vivo*. Targeted mutagenesis or ligand extension aimed at buttressing the PHE410 face should therefore block this escape hatch.

In summary, MM-PBSA paints a quantitatively coherent picture that aligns with the RMSD/SASA observables: amoxicillin relies on a deep electrostatic well buttressed by hydrophobic walls; resistance emerges when hinge dynamics momentarily erode one wall. Reinforcing vdW packing—either chemically *via* amoxicillin analogues or by optimizing structural stability—offers a rational route to reclaim β-lactam potency against multidrug-resistant *Salmonella typhimurium* (Fig. 11A–C and Table 2).

#### 3.2.4 Pharmacokinetic prediction and safety assessment of oleic acid-encapsulated amoxicillin nanoemulsion

In Table 3, The comprehensive pharmacokinetic and safety evaluation of amoxicillin encapsulated in oleic acid-based nanoemulsion reveals significant improvements in drug delivery properties and bioavailability compared to conventional amoxicillin formulations. Computational predictions demonstrate that the nanoemulsion system addresses the fundamental limitation of amoxicillin's poor oral bioavailability while maintaining an acceptable safety profile with enhanced therapeutic potential.

**Table 3:** Comparative pharmacokinetic and safety properties of amoxicillin and oleic acid nanoemulsion

Parameter	Amoxicillin	Oleic acid	Fold change	Clinical significance
Molecular weight (Da)	365.1	282.26	0.77	Smaller size may improve tissue penetration
Log *P*	−0.358	7.063	−19.7	Enhanced lipophilicity improves membrane permeation
TPSA (Ų)	132.96	37.3	0.28	Reduced polarity facilitates absorption
HIA (probability)	1.07 × 10^−6^	0.142	132 710	Dramatic improvement in absorption potential
Caco-2 permeability (log cm s^−1^)	−5.824	−5.081	5.5	Enhanced intestinal permeability
Oral bioavailability F30% (%)	0.0	72.2	∞	Transformation from non-bioavailable to highly bioavailable
PPB (%)	32.2	98.3	3.1	Sustained release potential
Half-life (h)	1.310	0.754	0.58	Faster elimination may require dosing optimization
DILI (probability)	0.853	0.009	0.01	95-Fold reduction in hepatotoxicity risk
Nephrotoxicity (probability)	0.872	0.390	0.45	Significant reduction in kidney toxicity
Ames mutagenicity (probability)	0.130	0.088	0.68	Improved genotoxicity profile
BBB penetration (probability)	0.000	0.018	18	Minimal CNS exposure maintaining safety

The molecular characterization reveals complementary properties between amoxicillin and oleic acid that support their synergistic combination in nanoemulsion formulations. Amoxicillin, with a molecular weight of 365.1 Da and topological polar surface area (TPSA) of 132.96 Ų, exhibits high polarity and extensive hydrogen bonding capacity (5 donors, 8 acceptors), while oleic acid demonstrates contrasting lipophilic characteristics with a molecular weight of 282.26 Da and TPSA of 37.3 Ų. The stark difference in rotatable bonds (amoxicillin: 5, oleic acid: 15) indicates that oleic acid provides significantly greater molecular flexibility, which enhances its ability to form stable nanoemulsion droplets and improve drug encapsulation efficiency. The Log *P* values further emphasize this complementarity, with amoxicillin's hydrophilic nature (Log *P*: −0.358) being balanced by oleic acid's extreme lipophilicity (Log *P*: 7.063), creating an optimal environment for enhanced membrane permeation and drug solubilization.

The most striking finding in the pharmacokinetic analysis is the dramatic improvement in predicted human intestinal absorption (HIA) for the nanoemulsion formulation. While amoxicillin alone shows virtually no intestinal absorption (HIA: 1.07 × 10^−6^), the oleic acid-encapsulated formulation demonstrates a substantial 132 000-fold increase in HIA probability (0.142). This remarkable enhancement correlates with improved membrane permeability characteristics, as evidenced by the Caco-2 permeability values showing amoxicillin at −5.824 log cm s^−1^ compared to oleic acid at −5.081 log cm s^−1^. The oral bioavailability predictions further support this improvement, with the nanoemulsion showing 28.6% probability at F20% threshold and 72.2% at F30% threshold, while amoxicillin consistently demonstrates zero bioavailability across all thresholds. These findings suggest that the nanoemulsion formulation could potentially transform amoxicillin from a poorly absorbed drug to one with clinically significant oral bioavailability.

The distribution characteristics reveal important differences that influence the therapeutic profile of the nanoemulsion system. Plasma protein binding (PPB) analysis shows a threefold increase for oleic acid (98.3%) compared to amoxicillin (32.2%), indicating that the nanoemulsion system may provide sustained drug release and prolonged therapeutic effects. The volume of distribution (VDss) remains comparable between compounds (−0.571 *vs.* −0.587 L kg^−1^), suggesting similar tissue penetration patterns. However, the plasma clearance rates differ moderately, with oleic acid showing slightly higher clearance (3.500 mL min^−1^ kg^−1^) compared to amoxicillin (3.010 mL min^−1^ kg^−1^). The half-life analysis reveals that oleic acid exhibits a shorter plasma residence time (0.754 h) compared to amoxicillin (1.310 h), which may necessitate optimization of dosing regimens to maintain therapeutic concentrations while leveraging the improved bioavailability.

The safety evaluation presents a markedly improved profile for the nanoemulsion formulation across multiple toxicological endpoints. The most significant improvement is observed in drug-induced liver injury (DILI) prediction, where oleic acid demonstrates a 95-fold reduction in hepatotoxicity risk (0.009) compared to amoxicillin's high DILI probability (0.853). This substantial decrease in predicted liver toxicity is particularly relevant given amoxicillin's known hepatotoxic potential in clinical practice. Genotoxicity assessment through Ames testing shows favorable results for both compounds, with oleic acid demonstrating a slightly lower mutagenic potential (0.088) compared to amoxicillin (0.130). Cardiotoxicity evaluation *via* hERG channel inhibition reveals acceptable risk levels for both compounds, with amoxicillin at 0.040 and oleic acid at 0.136 probability. The nephrotoxicity analysis indicates that while amoxicillin presents a concerning kidney toxicity risk (0.872), oleic acid shows a significantly reduced nephrotoxic potential (0.390), suggesting that the nanoemulsion formulation may mitigate renal adverse effects.

The blood–brain barrier (BBB) permeability analysis reveals minimal central nervous system penetration for both compounds, with amoxicillin showing negligible BBB crossing (0.000) and oleic acid demonstrating only minimal penetration (0.018). This limited CNS exposure is therapeutically advantageous for systemic antibiotic therapy, as it minimizes the risk of neurological side effects while maintaining systemic efficacy. The neurotoxicity predictions support this favorable CNS safety profile, with both compounds showing extremely low neurotoxic potential (amoxicillin: 0.007, oleic acid: 0.009).

The cytochrome P450 interaction analysis reveals minimal potential for drug–drug interactions in the nanoemulsion system. Both compounds demonstrate negligible CYP3A4 inhibition (amoxicillin: 0.005, oleic acid: 0.000) and substrate activity, indicating low risk for metabolic drug interactions. This favorable metabolic profile suggests that the nanoemulsion formulation would be suitable for use in polypharmacy situations without significant concerns about altering the pharmacokinetics of co-administered medications.

The comprehensive analysis demonstrates that oleic acid-based nanoemulsion encapsulation of amoxicillin represents a paradigm shift in the drug's pharmacokinetic and safety profile. The most remarkable finding is the transformation of amoxicillin from a poorly absorbed drug with significant toxicity concerns to a potentially highly bioavailable formulation with markedly improved safety characteristics. The 132 000-fold increase in predicted intestinal absorption, coupled with the 95-fold reduction in hepatotoxicity risk, suggests that this nanoemulsion system could address the fundamental limitations of conventional amoxicillin therapy.

The molecular complementarity between the hydrophilic amoxicillin and lipophilic oleic acid creates an optimal microenvironment for enhanced drug solubilization and membrane permeation. The high plasma protein binding of oleic acid (98.3%) may initially appear concerning; however, in the context of nanoemulsion drug delivery, this property can be advantageous by providing sustained drug release and prolonged therapeutic effects. The reduced half-life of the nanoemulsion system (0.754 h *vs.* 1.310 h) may necessitate dosing regimen optimization, but this is offset by the dramatic improvement in bioavailability.

Our simulations indicating membrane perturbation are consistent with literature demonstrating fatty-acid–induced outer- and inner-membrane damage. Standard assays (NPN uptake for OM, SYTOX Green/propidium iodide for IM) widely quantify such effects. Prior work shows that free fatty acids—including oleic acid—destabilize membranes, increase permeability, and can lead to leakage/lysis, supporting the mechanistic basis for our observed antibacterial effects.^95–98^

## 4. Conclusion

This study successfully demonstrates that formulating amoxicillin within an oleic acid nano-emulsion is a highly effective strategy for overcoming multidrug resistance in *Salmonella typhimurium*. The experimental results unequivocally show a more than two-fold enhancement in antibacterial activity, which is attributed to the favorable physicochemical properties of the nano-carrier that facilitate improved drug delivery and interaction with the bacterial cell.

The computational analysis provides a deep, mechanistic understanding of this enhanced efficacy. Molecular docking and dynamics simulations confirm that amoxicillin maintains a stable and high-affinity binding to its target, PBP3a, while the nano-carrier system fundamentally improves its pharmacokinetic and safety profile. The predicted increase in oral bioavailability and the substantial reduction in hepatotoxicity and nephrotoxicity address key limitations of conventional amoxicillin therapy.

In conclusion, the synergistic combination of experimental evidence and computational modeling provides a comprehensive validation of oleic acid nano-emulsions as a potent platform for reviving the clinical utility of conventional antibiotics against resistant pathogens. This work paves the way for the clinical translation of advanced drug delivery systems as a critical tool in the global fight against antimicrobial resistance. Future research should focus on *in vivo* efficacy studies and the optimization of this nano-formulation for clinical applications.

## 5. Study limitations

This work was designed to provide an initial, formulation-focused assessment using agar well-diffusion, which is qualitative; zone diameters are influenced by diffusion and matrix effects as well as antimicrobial potency. Consequently, we did not quantify activity by broth microdilution MIC/MBC or time-kill assays, and the present data should be interpreted as evidence of inhibition rather than absolute potency. Mechanistically, we did not perform direct membrane-permeability measurements (*e.g.*, NPN uptake for the outer membrane; SYTOX Green/propidium iodide for the inner membrane). Our molecular modeling supports plausibility but is limited to pre-acylation, non-covalent recognition of amoxicillin at PBP3; it does not simulate covalent acyl-enzyme formation, porin/efflux contributions, or the full periplasmic milieu.

On the physicochemical side, DLS/ζ-potential measurements were applied to the nanoemulsion droplets only; such analyses are not informative for free amoxicillin, a small, non-surface-active molecule. Electron microscopy of oil-in-water droplets is prone to low contrast and dehydration artifacts without negative stain or cryo-TEM; we therefore relied on DLS/ζ for primary sizing and provide standardized, high-resolution images for visualization. In addition, oleic-acid self-assembly is pH- and ionic-strength-dependent; while we discuss this behavior, we did not systematically map aggregation states across conditions, so our results are most applicable to the formulation environment tested.

Finally, the biological scope was limited to *Salmonella enterica* serovar *Typhimurium*; activity against other serovars or species remains to be determined. Cytotoxicity/hemolysis, *in vivo* pharmacology/toxicity, and formulation-level pharmacokinetics were not evaluated here. *In silico* ADMET outputs for individual molecules were used qualitatively and should not be interpreted as formulation bioavailability. Future work addressing these points will better define the potency, spectrum, safety, and translational potential of the amoxicillin–oleic-acid nanoemulsion.

## Abbreviation

AMR: Antimicrobial resistance
MDR: Multidrug-resistant
WHO: World Health Organization
GDP: Gross domestic product
CFU: Colony-forming unit
CLSI: Clinical & Laboratory Standards Institute
MIC: Minimum inhibitory concentration
β-Lactam: β-Lactam (four-membered cyclic amide antibiotic core)
GRAS: Generally Recognized As Safe (FDA excipient status)
NE: Nano-emulsion
PDI: Polydispersity index
DLS: Dynamic light scattering
TEM: Transmission electron microscopy
PBP: Penicillin-binding protein
PBP3a: Penicillin-binding protein 3a
FtsI: Filamentous temperature-sensitive protein I (gene encoding PBP3)
MD: Molecular dynamics
RMSD: Root-mean-square deviation
RMSF: Root-mean-square fluctuation
*R*_g_
: Radius of gyration
MM-PBSA/MMPBSA: Molecular Mechanics–Poisson–Boltzmann Surface Area (binding-energy method)
Δ*G*_bind_: Binding free energy change
CGenFF: CHARMM General Force Field
PME: Particle-mesh Ewald (long-range electrostatics algorithm)
TIP3P: Transferable Intermolecular Potential with 3 Points (water model)
ADMET: Absorption, Distribution, Metabolism, Excretion & Toxicity profiling
HIA: Human intestinal absorption
DILI: Drug-induced liver injury
BBB: Blood–brain barrier
PLIP: Protein–ligand interaction profiler
DSSP: Dictionary of secondary structure of proteins
PKA: cAMP-dependent protein kinase A
*R*
: The *R* statistical-computing language
RMSDbackbone: Backbone root-mean-square deviation
SD: Standard deviation

## Author contributions

S. E.: Conceptualized and executed the computational work (docking, MD simulations, MM-PBSA, ADMET) and drafted the computational sections. A. K. M., H. Y., H. A., E. M.: Performed nano-emulsion formulation, physicochemical characterization, antimicrobial assays, and experimental data analysis. M. W. S., W. M. E.: Designed experimental protocols, supervised formulation and microbiology work, and co-edited the experimental sections. A. M.: Provided overall project supervision, secured funding, coordinated efforts, and critically revised the manuscript.

## Conflicts of interest

The authors declare no competing interests.

## Data Availability

All data supporting the findings of this study are contained within the article. Additional details are available from the corresponding author upon reasonable request.
